# Hyperacute Rejection of a Living Unrelated Kidney Graft

**DOI:** 10.1155/2014/613641

**Published:** 2014-09-17

**Authors:** Dietlind Tittelbach-Helmrich, Dirk Bausch, Oliver Drognitz, Heike Goebel, Christian Schulz-Huotari, Albrecht Kramer-Zucker, Ulrich Theodor Hopt, Przemyslaw Pisarski

**Affiliations:** ^1^Department of General and Visceral Surgery, University of Freiburg, Hugstetter Straße 55, 79106 Freiburg im Breisgau, Germany; ^2^Department of General Surgery, University Hospital Lübeck, University of Lübeck, Ratzeburger Allee 160, 23538 Lübeck, Germany; ^3^Institute of Pathology, University of Freiburg, Breisacher Straße 115a, 79106 Freiburg im Breisgau, Germany; ^4^Department of Transfusion Medicine, University of Freiburg, Hugstetter Straße 55, 79106 Freiburg im Breisgau, Germany; ^5^Department of Nephrology, University of Freiburg, Hugstetter Straße 55, 79106 Freiburg im Breisgau, Germany

## Abstract

We present a case report of a 59-year-old man, who received a blood group identical living unrelated kidney graft. This was his second kidney transplantation. Pretransplant T-cell crossmatch resulted negative. B-cell crossmatch, which is not considered a strict contraindication for transplantation, resulted positive. During surgery no abnormalities occurred. Four hours after the transplantation diuresis suddenly decreased. In an immediately performed relaparotomy the transplanted kidney showed signs of hyperacute rejection and had to be removed. Pathological examination was consistent with hyperacute rejection. Depositions of IgM or IgG antibodies were not present in pathologic evaluation of the rejected kidney, suggesting that no irregular endothelial specific antibodies had been involved in the rejection. We recommend examining more closely recipients of second allografts, considering not only a positive T-cell crossmatch but also a positive B-cell crossmatch as exclusion criteria for transplantation.

## 1. Introduction

Hyperacute rejection is a rare event in kidney transplantation. It is mediated by preformed antibodies that can usually be excluded by a pretransplant CDC crossmatch. Origin of the circulating cytotoxic IgG HLA antibodies is most commonly a previous failed graft, blood transfusions, or pregnancy. Crossmatches are performed with unseparated cells, T-cells, or B-cells. A positive crossmatch with unseparated cells or T-cells alone is always a contraindication for transplantation, whereas the significance of a positive B-cell crossmatch remains unclear. However, there is growing evidence that there may exist hyperacute rejections mediated by endothelial, non-HLA antibodies that cannot be detected in standard T and B lymphocyte crossmatch techniques.

## 2. Case Report

### 2.1. Recipient

A 59-year-old male with end-stage renal disease due to nephrosclerosis was evaluated for a living unrelated kidney transplantation. A first kidney transplantation had been performed in 1993 (HLA of the first donor Table 1) and the kidney had functioned for 10 years. Graft failure was caused by chronic vascular rejection. The nonfunctioning kidney was removed shortly after its failure and the patient was treated again with hemodialysis. Blood group of the recipient was 0+, HLA type is shown in Table 1, and the most recent PRA level was 34%. The past medical history included hypertension, coronary heart disease, deep venous thrombosis, prolonged reversible ischemic neurologic deficit, and a central venous thrombosis of the right eye. Currently the patient was under anticoagulation with phenprocoumon. Pretransplant evaluation included negative studies for hepatitis B surface antigen, hepatitis B surface antibody, HIV antibody, hepatitis C antibody, CMV IgG, and CMV IgM. Induction and immunosuppression were started before surgery with basiliximab, tacrolimus, mycophenolate-mofetil, and prednisone. The patient received a living unrelated kidney graft from his wife. Pretransplant T lymphocyte crossmatch was negative, whereas the B-cell crossmatch was positive.

### 2.2. Donor

**Figure 1:**
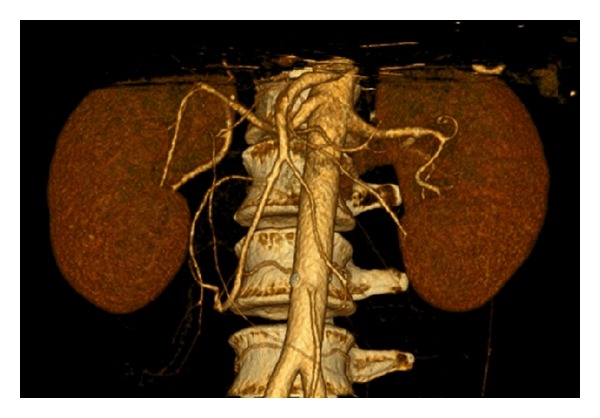
Angio-computed tomography of the kidneys of the 2nd donor: on both sides single strong renal artery without signs of kidney pathologies.

A 53-year-old nonrelative female, blood type 0+, HLA shown in Table 1. There was no medical history or regular medication. Both kidneys had a normal anatomy with each a single artery and vein (Figure 1). The creatinine clearance calculated by Cockroft and Gault was 80 mL/h. Since the left kidney had a partial function of 47% it was chosen for donation. Nephrectomy was uneventful. Perfusion was performed with HTK-solution and did not present any abnormalities.

### 2.3. Post-Transplant

**Figure 2:**
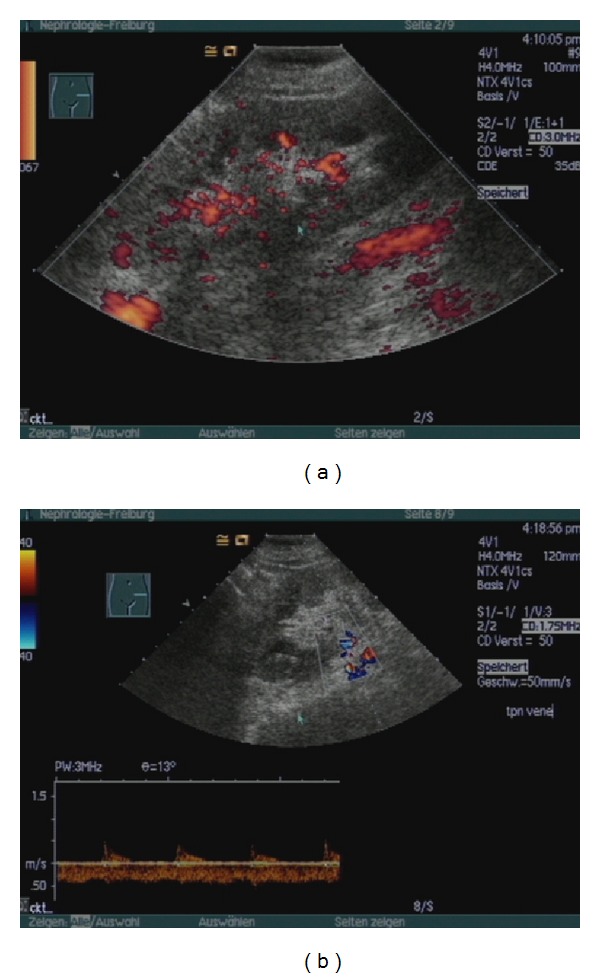
Duplex ultrasonography of the transplanted kidney 3 hs after transplantation; free perfusion of vein and artery, peripheral perfusion deficit, high resistance indices, and prominent pyramids are characteristic for acute renal failure.

**Figure 3:**
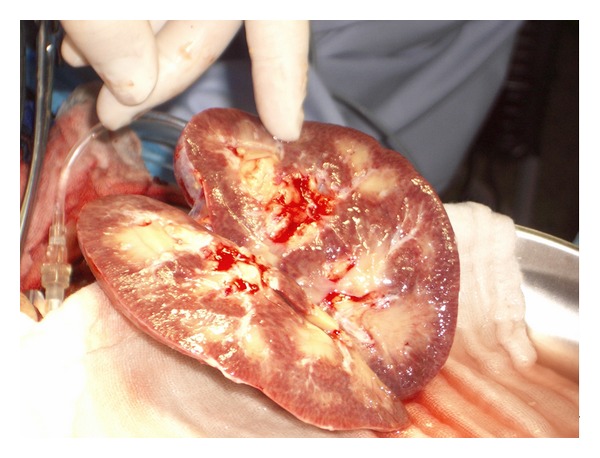
Macroscopic appearance of the rejected kidney during relaparotomy.

After a cold ischemia time of 143 min the transplantation was performed on the left iliac axis with vascular anastomosis between the renal artery and the common iliac artery and renal vein and external iliac vein. Bladder anastomosis was performed in a modified Lich-Gregoir technique. Immediately after restoration of blood flow urine production was observed. The kidney had a normal aspect with a good turgor. The transplantation was uneventful. During the first three hours following the surgery urine production continued with a volume of about 150 mL per hour. Then urine production suddenly decreased. General symptoms did not occur and the patient remained cardiorespiratory stable. An immediately performed duplex scan revealed a normal perfusion of the renal artery and vein, but there were signs of acute renal failure present (Figure 2). Differential diagnostic included acute humoral rejection, acute tubular necrosis, and a thromboembolic event. During relaparotomy the transplanted kidney impressed edematous, flaccid, and cyanotic (Figure 3). It had to be removed and the patient continued with hemodialysis. The postoperatively repeated T- and B-cell crossmatch resulted negative.

### 2.4. Renal Pathology

**Figure 4:**
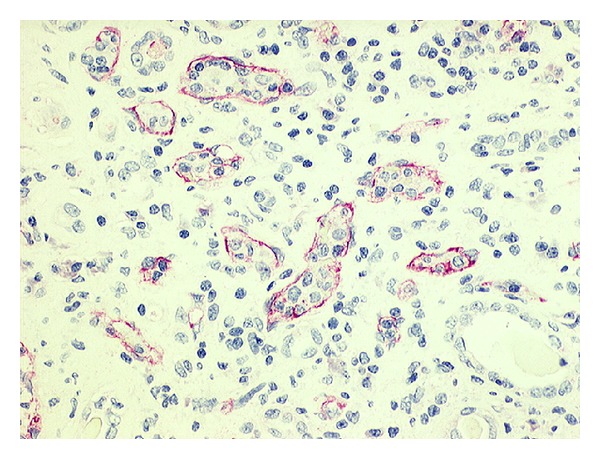
Attachment of C4d along the peritubular capillaries.

In the histologic examination of the explanted kidney there was no hint for thromboembolic events of larger vessels. However capillary lumina were partially occluded by erythrocytes, granulocytes, and thrombocytes and adherent leucocytes near to or in the subendothelial surface were present. Fluorescent study did not show any immunoglobulin or complement, while there was a diffuse attachment of C4d along the peritubular capillaries (Figure 4).

In summary histologic examination of the graft was consistent with a hyperacute rejection.

## 3. Discussion

In our days hyperacute rejection is an uncommon event, due to preformed cytotoxic antibodies which can normally be excluded by a negative pretransplant crossmatch. A strict contraindication for transplantation is a positive T-cell crossmatch, the clinical significance of an isolated positive B-cell crossmatch, detecting HLA class II antibodies and weak class I antibodies is currently no well defined [1].

In our patient the crossmatch was performed three times. T-cell crossmatches always resulted negative, whereas B-cell crossmatches were positive in two of three repetitions. At the time of transplantation PRA level was 34%. The antibodies were specific for HLA A32 which was not present in the second donor; therefore it was decided to perform the transplantation despite a positive B-cell crossmatch. Immediately after the resurgery the crossmatch was repeated, but resulted negative. Blood samples taken three months later were tested positive for anti-HLA I and HLA II antibodies and on unseparated cells and B- and T-lymphocytes. The antibodies were directed against the HLA antigens of the actually and previously failed grafts, but there was also unspecific activity present. There is accumulating evidence that there exist antigenic systems not detectable in standard lymphocytotoxic techniques that may provoke hyperacute or accelerated rejection: the endothelial-monocyte alloantigen system [2], the anti-vascular IgG endothelial cell antibodies [3], and the IgM antibodies that are reactive against endothelial cells [4]. So it remains unclear whether the hyperacute rejection was caused by preformed antibodies against present HLA antigens (e.g., HLA DR9 that were expressed both in the first and in the second donor) or there were other non-HLA antibodies reacting with the endothelium, although this possibility seems rather unlikely considering the absence of depositions of IgM or IgG in the histological findings.

In addition to that, it has been shown that activated protein C resistance and Factor V mutation are associated with an increased incidence of acute rejection and graft loss in kidney transplant recipients [5–7]. Although there was no such mutation proven in our patient, he had suffered from several thromboembolic events which required a continuous anticoagulation. Ten days prior to the hospital admission medication with phenprocoumon was discontinued and replaced by low-molecular weight heparin. It remains unclear whether this prothrombotic state has also contributed to the graft loss.

Another important issue is how to proceed in the future with possibly offered organs. Surely the patient has an increased risk for rejection and loss of a third kidney graft, but the question is how to minimize this risk. We now listed him again for kidney transplantation. The HLA antigens A2, A32, B18, B44, B60, Cw7, Cw3, Cw5, DR9, DR13, DQ2, DQ3, and DQ6 (antigens of former donors and proven antibody specificities) were given as unacceptable antigens. The immunosuppression for a third kidney transplantation needs to be intensified by ATG or other depleting antibodies for induction and, possibly, with an earlier start of the immunosuppression analogous to the ABO-incompatible protocol.

In general a positive pretransplant B-cell crossmatch might be considered a contraindication and establishing a wider spectrum of pretransplant tests would be useful in preventing hyperacute rejections in patients receiving a second kidney graft.

## Figures and Tables

**Table 1:** HLA-matches of recipient and 1st and 2nd donor.

Recipient		A3	A11		B5		B35		B51(5)		Bw4	Bw6	Cw1		Cw4			DR1					DQ1			DQ5	

(1) Donor		A3		A19(32)	B5(51)	B18					Bw4	Bw6					Cw7	DR1		DR9		DR53	DQ1		DQ3		

(2) Donor	A2							B44(12)		B60(49)				Cw3		Cw5		DR1	DR3	DR9	DR13			DQ2			DQ6
